# Supplementary material for: Delineating cooperative effects of Notch and biomechanical signals on patterned liver differentiation
Source: Commun Biol. 2022 Oct 7;5:1073. doi: 10.1038/s42003-022-03840-9 (PMC9546876; doi:10.1038/s42003-022-03840-9)
Supplement: Supplementary file 2 — Supplementary Information [file 42003_2022_3840_MOESM2_ESM.pdf]

Parameter values in the base model	Value
BetaN, Notch Receptor production	2
BetaNICD, NICD production	2
BetaD, Dll1 production	20
BetaJ, Jag1 production	20
k_{tD}, DLL1 mediated transactivation strength	0.3
k_{tJ}, JAG1 mediated transactivation strength	22
k_{tC}, DLL1 mediated cis-inhibition strength	10

Supplemental Table 1: Parameter values and explanation for the Notch signaling computational modeling.

Parameters values in different perturbations	Value
betaD, simulation for Dll1 KD	5
betaJ, simulation for Jag1 KD	5
ECADp, Simulation for ECad KD	0.3
Egf, simulate EGF treatment	100
tgfD, TGFb2 influence on DLL1 production	0.4
tgfJ, TGFb2 influence on JAG1 production	0.4
tgf, TGFb2 mediated expression of Notch targets	0.001
v, strength of E-Cad affecting TGFb effect	5

Supplemental Table 2: Parameter values that were used for various perturbations

Initial Conditions	Value
Dll1 [t=0]	$D_{0i} = 0.02 * betaD(1 + 0.2 * U_i)$ where U_i= random number between (-0.5,0.5)
Jag1 [t=0]	$J_{0i} = 0.02 * betaJ(1 + 0.2 * U_i)$ where U_i= random number between (-0.5,0.5)
NICD [t=0]	0
Notch Receptor [t=0]	betaN
Notch Targets [t=0]	0

Supplemental Table 3: Initial conditions for the Notch ligand, receptor, NICD and Notch target in the computational model.

Primer	Sequence (5’-3’)
Cadherin 1 Forward	TCGTTGTTGTCACAGACCCC
Cadherin 1 Reverse	GCTGCTTGGCCTCAAAATCC
DLL1 Forward	ACGGAGAAGGTTGCTCTGTGTTCT
DLL1 Reverse	CACTCCCCTGGTTTGTCACAGTAT
JAG1 Forward	CCGTAATCGCATCGTACTGCCTTT
JAG1 Reverse	ATTGCCGGCTAGGGTTTATCATGC
HPRT Forward	GGGACGCAGCAACTGACATTTCTA
HPRT Reverse	GGAGTCCTGTTGATGTTGCCAGTA

Supplemental Table 4: PCR Primer sequences

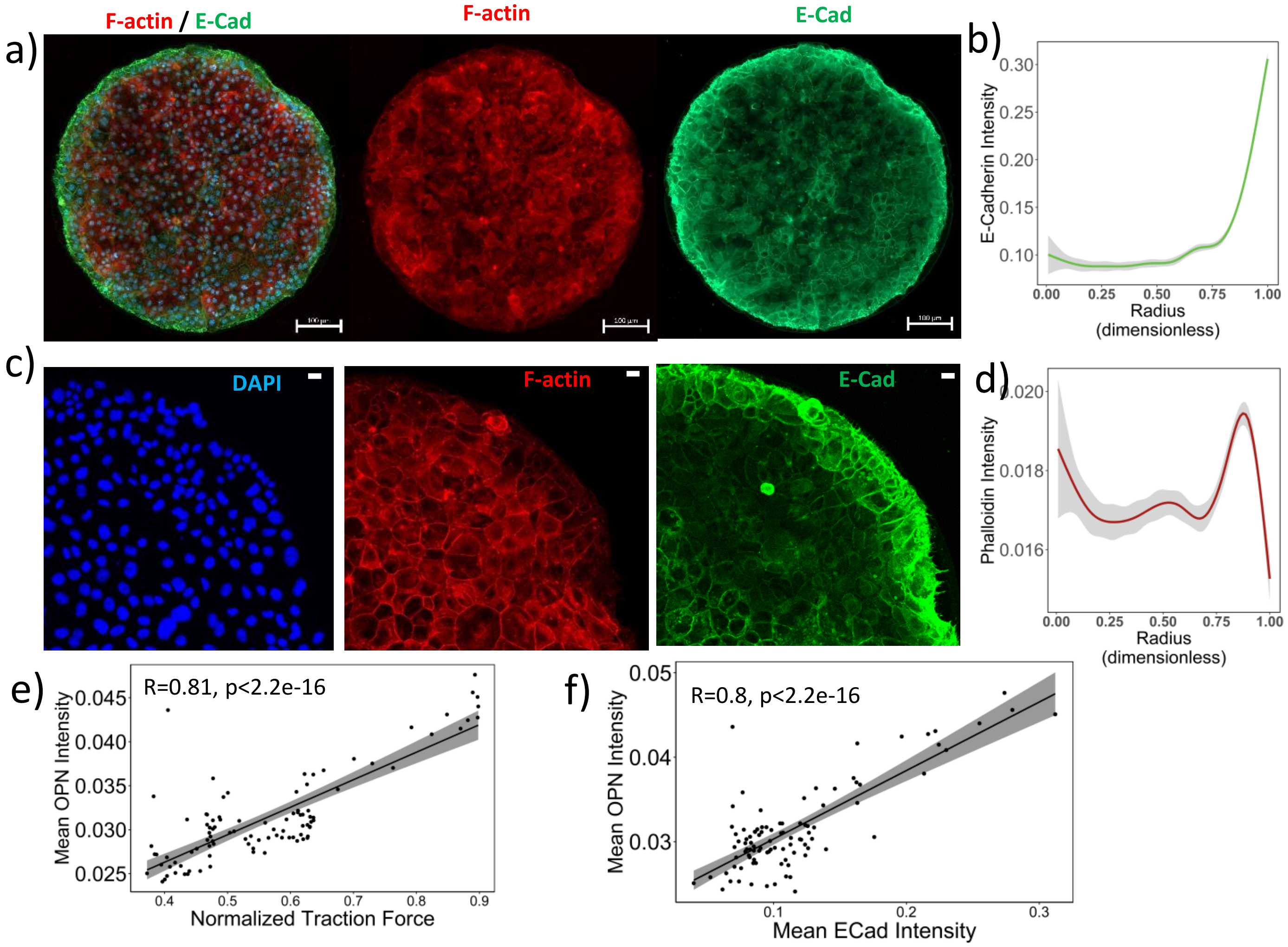

Supplemental Figure 1: E-Cadherin and F-actin patterning: a) Representative fluorescent Images of BMELs on Col 1 microarrays on 25kPa polyacrylamide hydrogels. Red: F-actin, Green: E-Cadherin, Blue: DAPI. Scale Bar: 100 microns. b,d) Quantification of mean E-Cadherin and F-actin Intensity/cell/island as a function of the radius. 0 is center, 1 is the edge. Grey: 95% confidence interval. n= 3 biological replicates (independent experiments) and >= 10 technical replicates (individual islands) c) Representative confocal maximum intensity projection images of BMELs on Col 1 microarrays on 25kPa polyacrylamide hydrogels. Red: F-actin, Green: E-Cadherin, Blue: DAPI. Scale Bar: 20 microns. Image taken with 20x objective. e,f) Correlation plots for correlation of Normalized Traction Force and Mean Ecad Intensity/Cells on an island to Mean OPN Intensity/Cell on an island. Pearson Correlation Coefficient is displayed on each graph as the correlation metric.

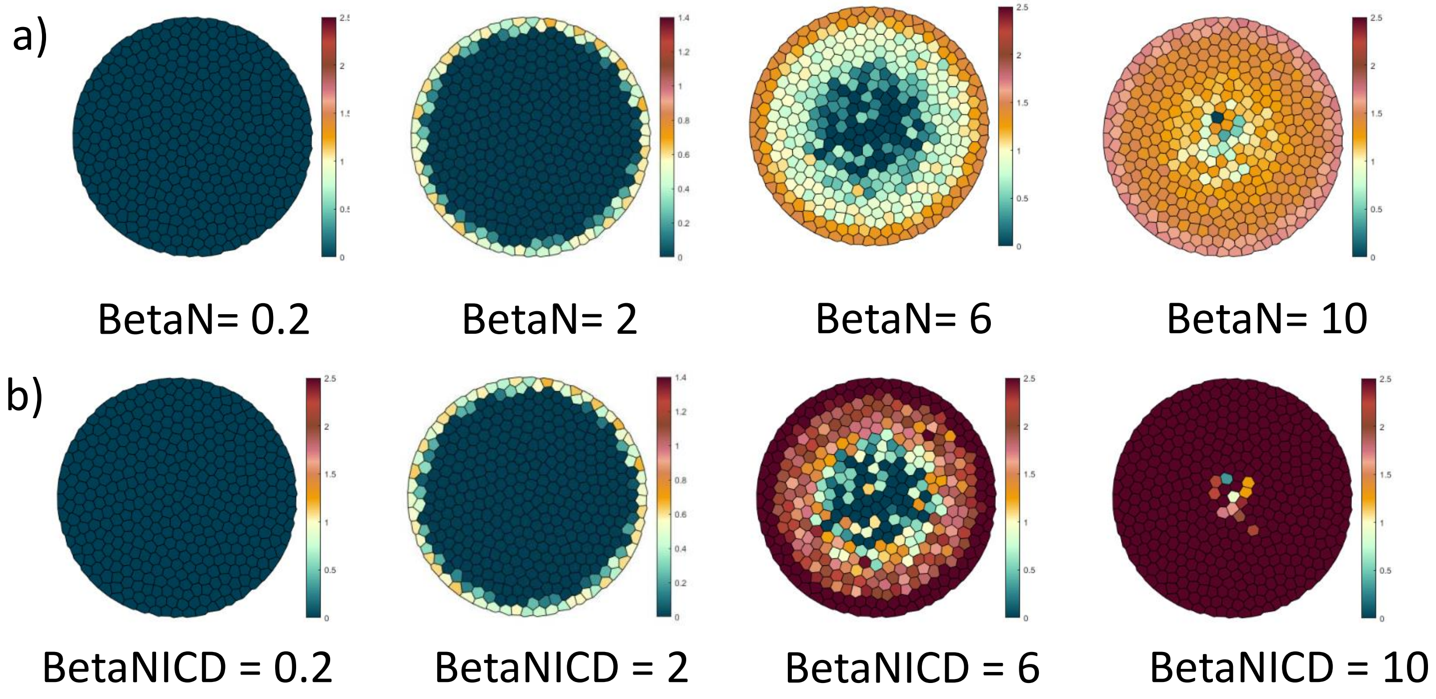

Supplemental Figure 2: Parameter Optimization for the computational model. a) Effect of modulating the parameter value for the expression of the Notch receptor, BetaN. In the model, 2 value is used for the all the perturbations. b) Effect of modulating the parameter value for the cleavage effect of the NICD, BetaNICD. In the model, 2 value is used for the all the perturbations.

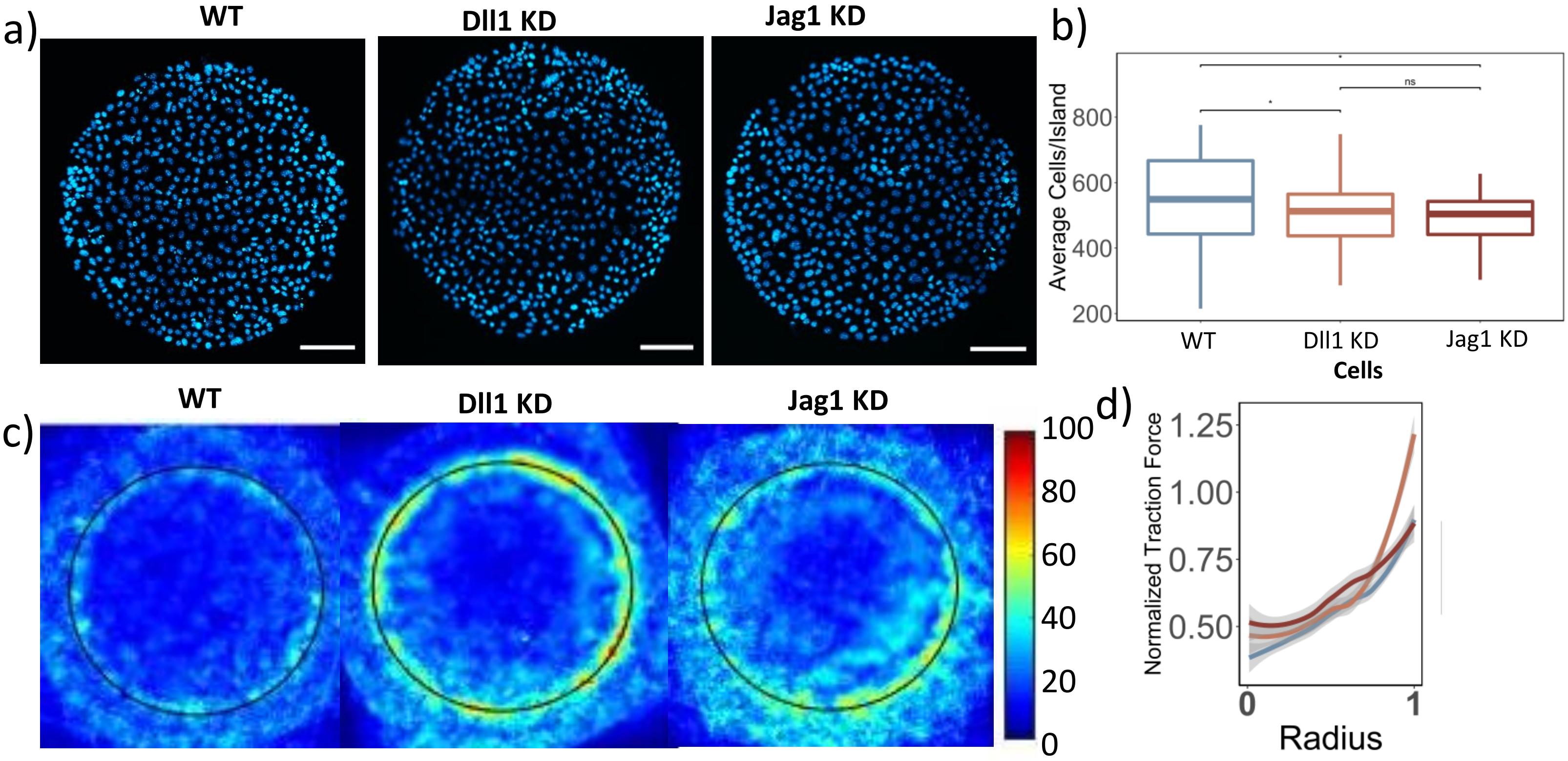

Supplemental Figure 3: Average Cell Number and Traction Force Quantification of BMELs with Notch ligand knockdowns on circular microarrays. a) DAPI fluorescent Images for WT, Dll1 KD and Jag1 KD BMELs on circular microarrays. Scale Bar: 100 microns. b) Quantification of Average Cell Number per individual island for WT, Dll1 KD and Jag1 KD BMELs. Boxplots- 'ns': ns; '*': p-value <0.05; '**': p-value <0.01; '***': p-value<0.001 '****': p-value<0.0001, calculated using Wilcox test in R. n>= 4 biological replicates (independent experiments) and n>= 20 technical replicates (individual islands). c) Traction force heatmap (Pa) for WT, Dll1 KD and Jag1 KD BMELs on circular islands, blue (low traction) to red (high traction). Scale: Pa d) Normalized Traction Force of BMELs as function of the radius, 0 being the center and 1 being the edge. Grey: 95% confidence interval. n>= 4 biological replicates (independent experiments) and n>= 15 technical replicates (individual islands).

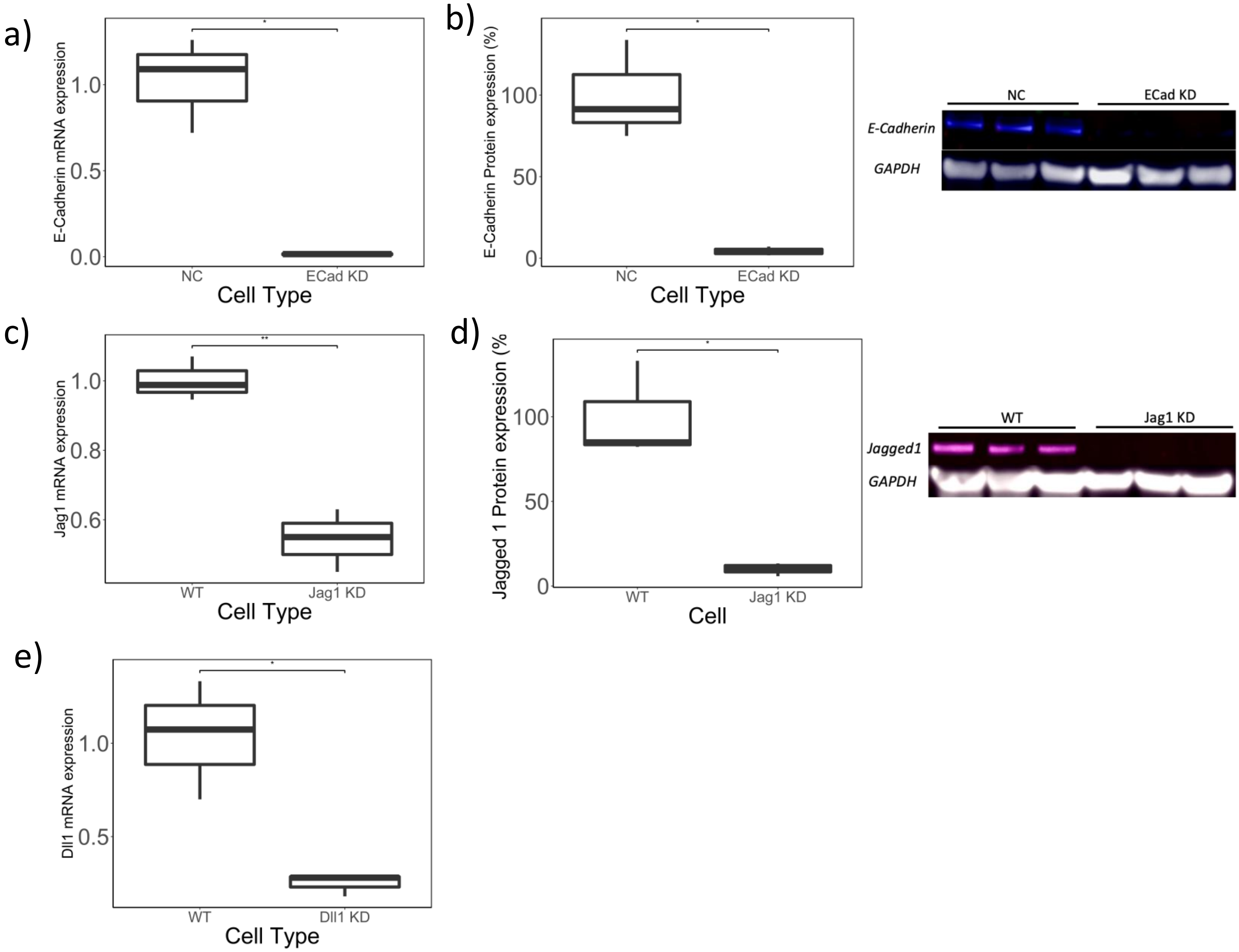

Supplemental Figure 4: RT-PCR and Western Blot quantification of the gene knockdowns in BMELs a) RT-PCR quantification E-Cadherin gene in BMELs with E-Cad knockdown by lipid transfection of siRNA b) Western Blot Quantification of E-Cadherin protein at 120kDa in BMELs with E-Cad knockdown by lipid transfection of siRNA. 3 biological replicates shown for NC and ECad KD. The BMELs were treated with Nonsense siRNA as the negative control (NC) and siRNA against Cadherin-1 mRNA (ECad KD) for 24 hours on Tissue Culture Plastic, prior to collection of sample for RT-PCR and Western Blot c) RT-PCR Quantification of Jagged1 gene in BMELs with Jag1 knockdown by lentiviral transduction of shRNA. d) Western Blot Quantification of Jagged1 protein in BMELs with Jag1 knockdown. 3 biological replicates shown for WT and Jag1 KD. e) RT-PCR Quantification of Dll1 gene in BMELs Jag1 knockdown by lentiviral transduction of shRNA. All mRNA expression values are normalized to HPRT gene for each sample and then normalized to Control cells for each knockdown. All protein expression values are normalized to GAPDH protein for each sample and then normalized to Control cells for each knockdown.

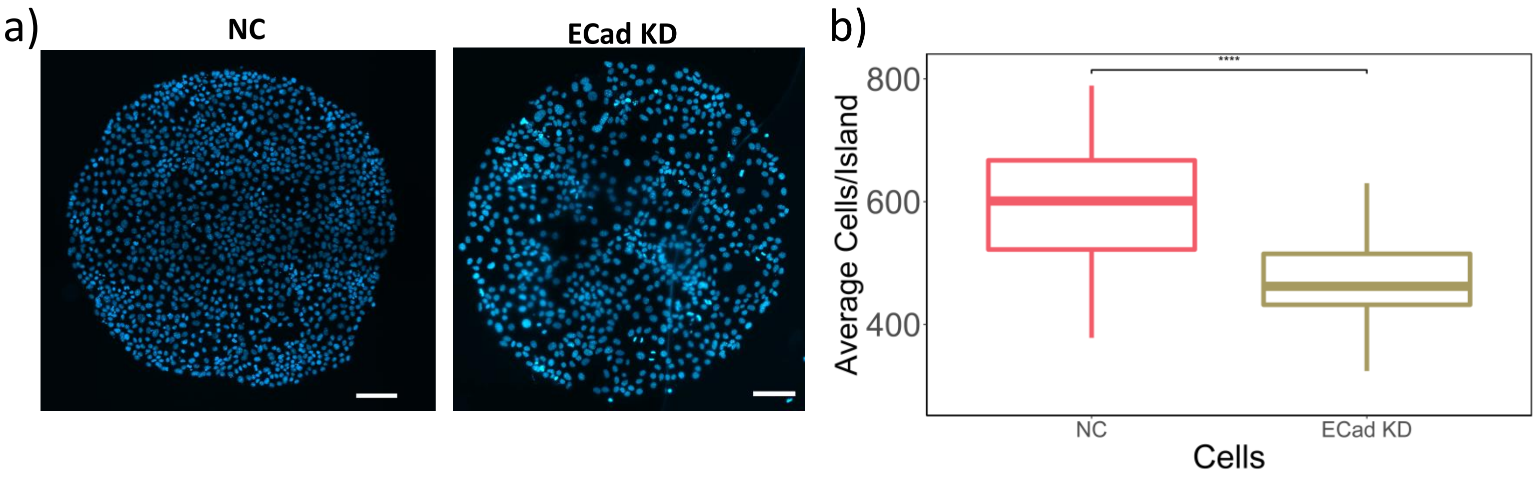

Supplemental Figure 5: a) DAPI fluorescent Images for NC and ECad KD BMELs on circular microarrays. Scale Bar: 100 microns. b) Quantification of Average Cell Number per individual island for WT, DLL1 KD and JAG1 KD BMELs. '****': p-value<0.0001, calculated using Wilcox test in R. n>= 4 biological replicates (independent experiments) and >= 20 technical replicates (individual islands)

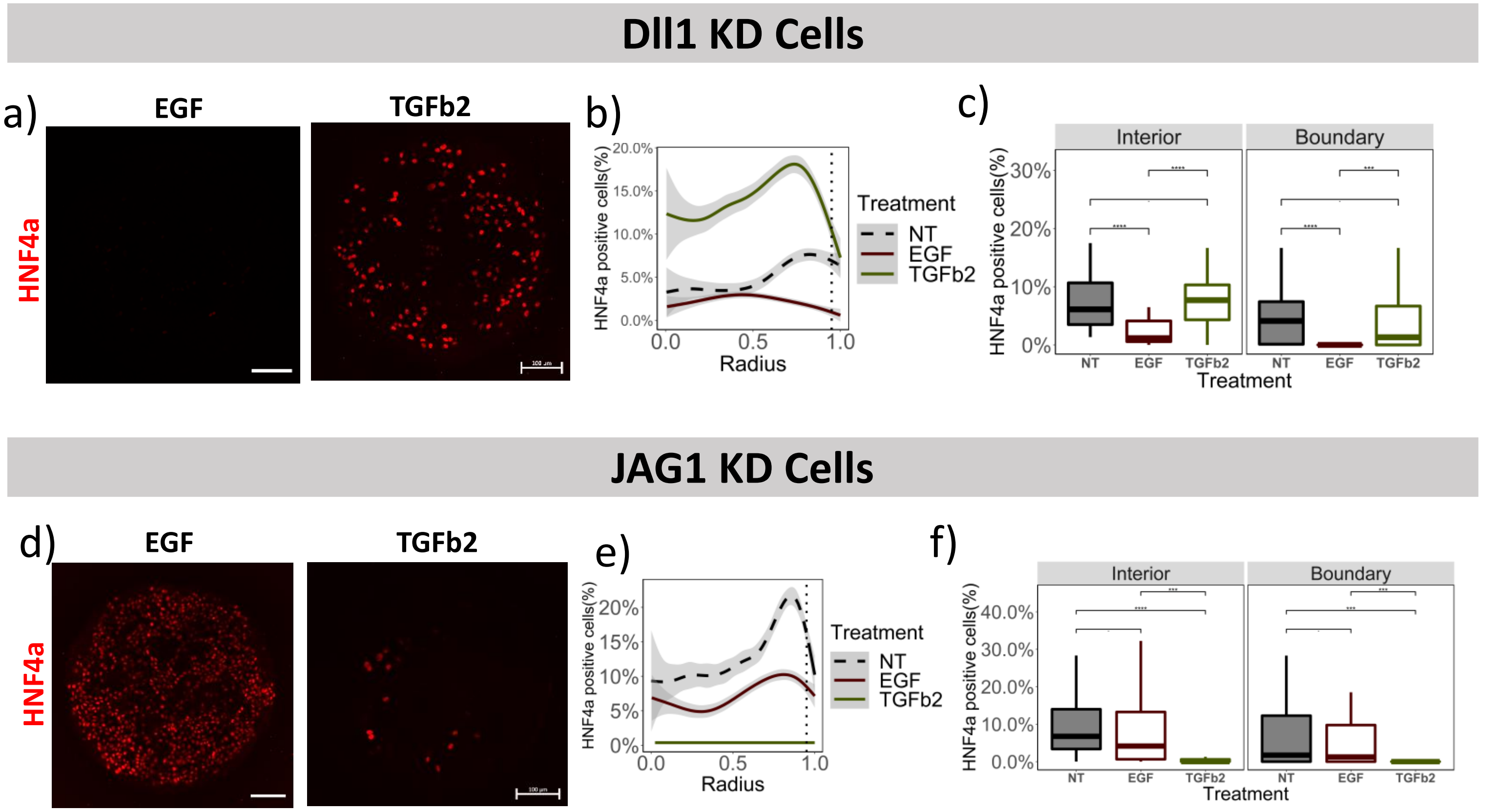

Supplemental Figure 6: HNF4a Quantification with growth factor treatment and Notch ligand knockdown. a,d) Representative fluorescent Images of EGF and TGFb2 treated Dll1 KD and Jag1 KD BMELs on Col 1 microarrays on 25kPa polyacrylamide hydrogels. Red: HNF4a, Blue: DAPI. Scale Bar: 100 microns. b,e) Quantification of average percent HNF4a+ cells/island as a function of the radius. 0 is center, 1 is the edge. n>= 4 biological replicates (independent experiments) and n>= 20 technical replicates (individual islands) c,f) Quantification of average percent HNF4a+ cells/island in the interior (radius = 0 – 0.95) and at the boundary (radius= 0.95 – 1.00). Boxplots- ‘.’: ns; ‘*’: p-value <0.05; ‘**’: p-value <0.01; ‘***’: p-value<0.001 ‘****’: p-value<0.0001, calculated using Wilcox test in R. Line plots- Grey: 95% confidence interval. n>= 4 biological replicates (independent experiments) and >= 20 technical replicates (individual islands) . The No Treatment Control (NT) for each knockdown is referred from Fig 3 b,c for statistical comparison.

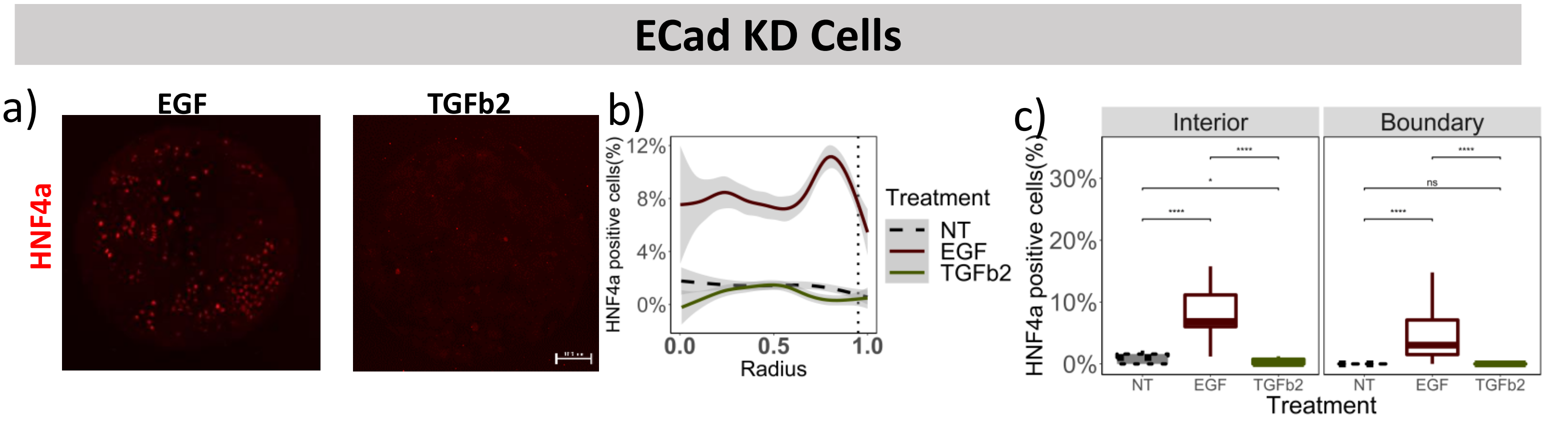

Supplemental Figure 7: HNF4a Quantification with growth factor treatment and E-Cadherin knockdown. a) Representative fluorescent Images of EGF and TGFb2 treated ECad KD BMELs on Col 1 microarrays on 25kPa polyacrylamide hydrogels. Red: HNF4a Scale Bar: 100 microns. BbQuantification of average percent HNF4a+ cells/island as a function of the radius. 0 is center, 1 is the edge. n>= 4 biological replicates (independent experiments) and n>= 20 technical replicates (individual islands) c) Quantification of percent HNF4a+ cells in the interior (radius = 0 – 0.95) and at the boundary (radius= 0.95 – 1.00). Boxplots- ‘.’: ns; ‘*’: p-value <0.05; ‘**’: p-value <0.01; ‘***’: p-value<0.001 ‘****’: p-value<0.0001, calculated using Wilcox test in R. Line plots- Grey: 95% confidence interval. n>= 4 biological replicates (independent experiments) and n>= 20 technical replicates (individual islands). The No Treatment Control (NT) for ECad KD knockdown is referred from Fig 4 b,c for statistical comparison.

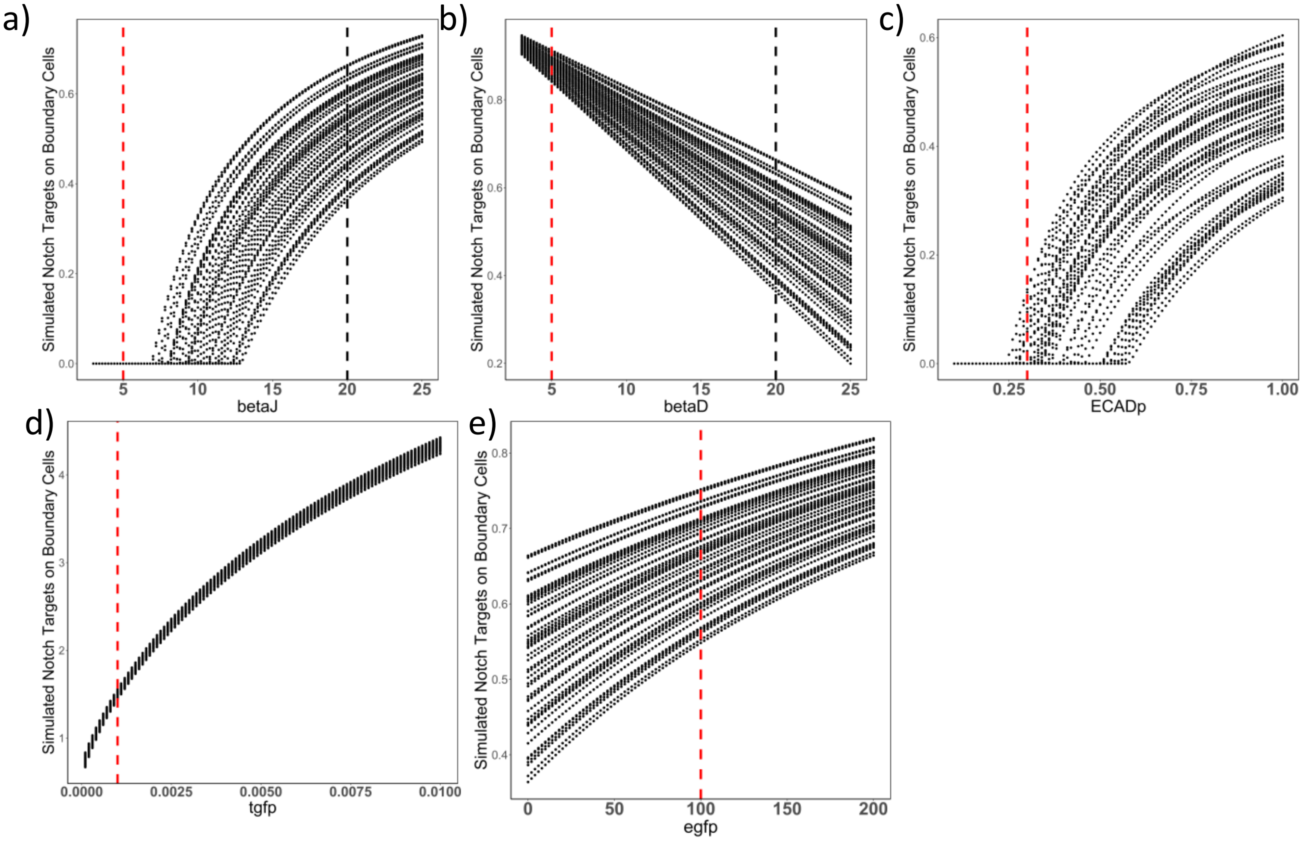

Supplemental Figure 8: Sensitivity analysis of the parameter used for the various in silico knockdown and growth factor perturbations. For the output metric when each parameter was perturbed across a range, the value of simulated notch targets in all the boundary cells (r>=0.95, 50 cells in the model) is displayed in each graph. A single point in the graph represent a single cell where the value of the parameter is on the x-axis and the result simulated notch targets is on the y-axis. a) Value of simulated Notch targets on the boundary cells when the parameter betaJ (Jag1 production) was perturbed from 3 to 25, with 0.2 increments. For Jag1 knockdown simulation, the value of betaJ = 5 was chosen (red line), whereas the base model betaJ = 20(black line). b) Value of simulated Notch targets on the boundary cells when the parameter betaD (Dll1 production) was perturbed from 3 to 25, with 0.2 increments. For Dll1 knockdown simulation, the value of betaD = 5 was chosen (red line), whereas the base model betaD = 20(black line). c) Value of simulated Notch targets on the boundary cells when the parameter for ECad intensity (ECADp, data from Supp Fig 1) was perturbed from 0.1 to 1, with 0.01 increments. In the base model ECADp varies as a function of radius based on our experimental observation and normalized to the maximum intensity which was measured at the edge. For the ECad knockdown simulation has a constant value of 0.3 across the whole island (red line). d) Value of simulated Notch targets on the boundary cells when the parameter egfp (EGF treatment parameter) was perturbed from 0 to 200, with increments of 2. For EGF knockdown simulation, the value of egfp = 100 was chosen (red line), whereas the base model had egfp=0. e) Value of simulated Notch targets on the boundary cells when the parameter tgfp (TGFb2 treatment parameter) was perturbed from 0.0001 to 0.01, with increments of 0.0001. For TGFb2 knockdown simulation, the value of tgfp = 0.001 was chosen (red line), whereas the base model had tgfp=0.

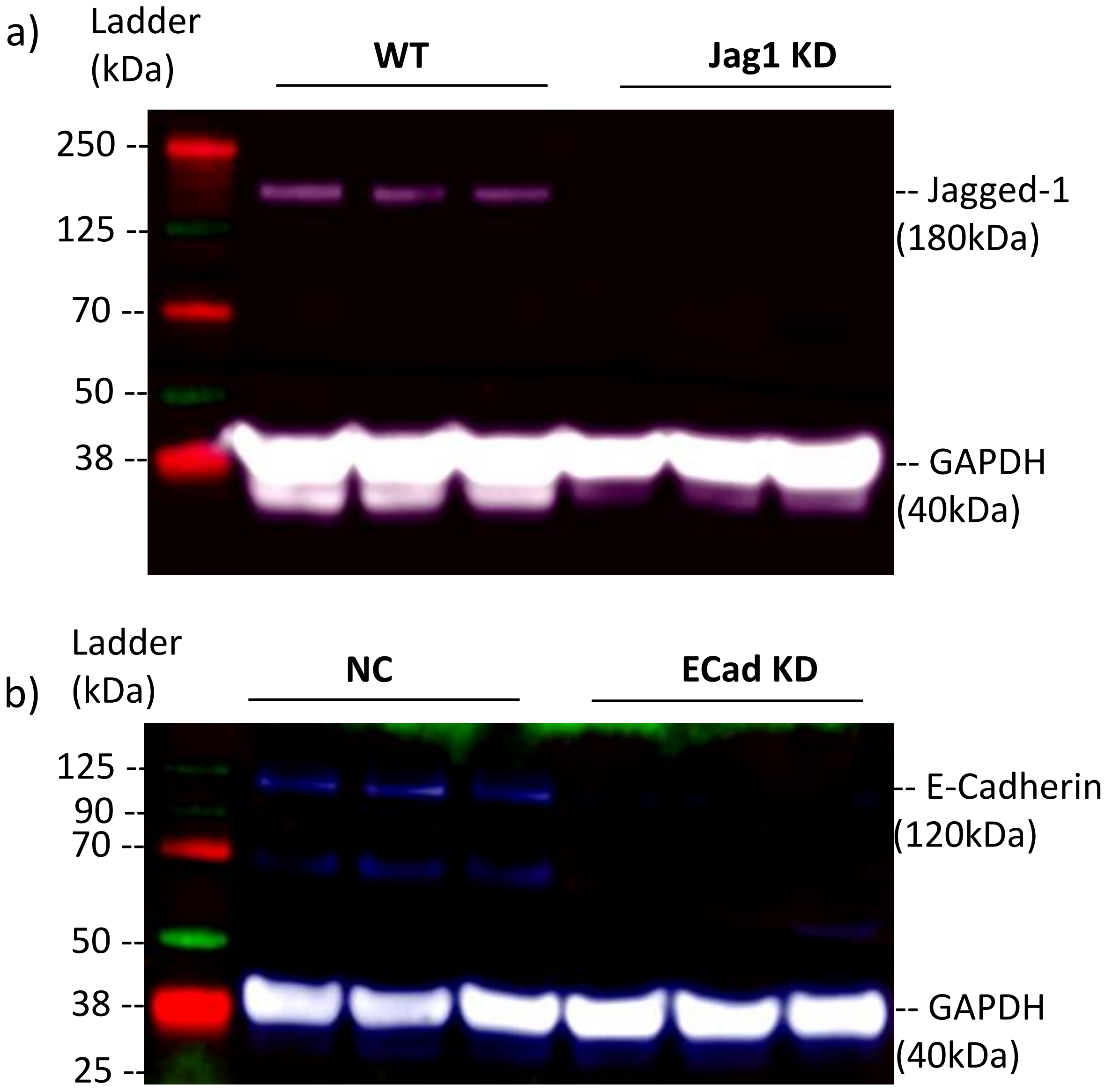

Supplemental Figure 9: Unedited/uncropped western blot gel images, quantification shown in Supplemental Figure 4. a) Western Blot gel images for Jagged-1 protein quantification for Wild Type (WT) and Jag1 KD BMELs. Three biological replicates are displayed for WT and Jag1 KD. b) Western Blot gel image for Jagged-1 protein quantification for Negative Control (NC) and Ecad KD BMELs. Three biological replicates are displayed for NC and Ecad KD. The first lane is WesternSure Pre_Stained Chemiluminiscent Protein Ladder, with size labels.
