# Supplementary material for: Delineating cooperative effects of Notch and biomechanical signals on patterned liver differentiation
Source: Commun Biol. 2022 Oct 7;5:1073. doi: 10.1038/s42003-022-03840-9 (PMC9546876; doi:10.1038/s42003-022-03840-9)
Supplement: Supplementary file 3 — Description of Additional Supplementary Files [file 42003_2022_3840_MOESM3_ESM.pdf]

Description of Additional Supplementary Files

File name: Supplementary Data 1
Description: The raw experimental data that used to make graphs in the paper.
