# Supplementary material for: Delineating cooperative effects of Notch and biomechanical signals on patterned liver differentiation
Source: Commun Biol. 2022 Oct 7;5:1073. doi: 10.1038/s42003-022-03840-9 (PMC9546876; doi:10.1038/s42003-022-03840-9)
Supplement: Supplementary file 5 — Reporting Summary [file 42003_2022_3840_MOESM5_ESM.pdf]

nature portfolio

Corresponding author(s): Dr. Gregory Underhill

Last updated by author(s): Jun 22, 2022

Reporting Summary

Nature Portfolio wishes to improve the reproducibility of the work that we publish. This form provides structure for consistency and transparency in reporting. For further information on Nature Portfolio policies, see our Editorial Policies and the Editorial Policy Checklist.

Statistics

For all statistical analyses, confirm that the following items are present in the figure legend, table legend, main text, or Methods section.

n/a	Confirmed	
☐	☒	The exact sample size (*n*) for each experimental group/condition, given as a discrete number and unit of measurement
☐	☒	A statement on whether measurements were taken from distinct samples or whether the same sample was measured repeatedly
☐	☒	The statistical test(s) used AND whether they are one- or two-sided<br>*Only common tests should be described solely by name; describe more complex techniques in the Methods section.*
☒	☐	A description of all covariates tested
☒	☐	A description of any assumptions or corrections, such as tests of normality and adjustment for multiple comparisons
☐	☒	A full description of the statistical parameters including central tendency (e.g. means) or other basic estimates (e.g. regression coefficient) AND variation (e.g. standard deviation) or associated estimates of uncertainty (e.g. confidence intervals)
☐	☒	For null hypothesis testing, the test statistic (e.g. *F*, *t*, *r*) with confidence intervals, effect sizes, degrees of freedom and *P* value noted<br>*Give P values as exact values whenever suitable.*
☒	☐	For Bayesian analysis, information on the choice of priors and Markov chain Monte Carlo settings
☒	☐	For hierarchical and complex designs, identification of the appropriate level for tests and full reporting of outcomes
☒	☐	Estimates of effect sizes (e.g. Cohen's *d*, Pearson's *r*), indicating how they were calculated

Our web collection on statistics for biologists contains articles on many of the points above.

Software and code

Policy information about availability of computer code

Data collection	Matlab 2018b is used for traction force microscopy and computational modeling of Notch signaling. The code for traction force microscopy is referenced in the Methods section and the custom code for computational modeling is available in the box folder: https://uofi.box.com/s/m37h5k82x9rdm9ansrhv9z792vegucq8
Data analysis	Cell Profiler 4.2.0 is used to analyze all the fluorescent images. RStudio Version 1.3.959 is used to analyze data obtained from Cell Profiler, statistical tests, plotting the graphs.

For manuscripts utilizing custom algorithms or software that are central to the research but not yet described in published literature, software must be made available to editors and reviewers. We strongly encourage code deposition in a community repository (e.g. GitHub). See the Nature Portfolio guidelines for submitting code & software for further information.

Data

Policy information about availability of data

All manuscripts must include a data availability statement. This statement should provide the following information, where applicable:

- Accession codes, unique identifiers, or web links for publicly available datasets
- A description of any restrictions on data availability
- For clinical datasets or third party data, please ensure that the statement adheres to our policy

Data outputs from cell microarrays and distinct model configurations are available through this link: https://uofi.box.com/s/a3h0dm74zl8f18v1zzmer1fqhapskmfz

Field-specific reporting

Please select the one below that is the best fit for your research. If you are not sure, read the appropriate sections before making your selection.

☒ Life sciences ☐ Behavioural & social sciences ☐ Ecological, evolutionary & environmental sciences

For a reference copy of the document with all sections, see nature.com/documents/nr-reporting-summary-flat.pdf

Life sciences study design

All studies must disclose on these points even when the disclosure is negative.

Sample size	Sample size >= 3 independent biological replicates and sample size >= 15 were used based on standard norms in the in vitro cell-based research.
Data exclusions	No data exclusions were performed
Replication	Multiple independent biological experiments were performed and analyzed. The differences were then evaluted for thier statistical significance among all the replicates. Each biological replicate had multiple technical replicates to capture the whole distribution of the population.
Randomization	The experimental groups for our studies is a single coverslip with a polyacrylamide hydrogel with 9 cellular microarrays on them. Each of the coverslip has a specific gene knockdown or no knockdown and growth factor treatment or no growth factor treatment. The order in which these specific conditions were applied to each coverslip was random across all experiments.
Blinding	The process of data collection was via automated imaging using a microscope, automated cell segmentation using Cell Profiler and then analysis of data in RStudio. Human intervention for data collection/analysis was only for quality control steps such as verifying that images of the microarrays were not cut in the middle, or chipped off anywhere, hence it wasn't blinded.

Reporting for specific materials, systems and methods

We require information from authors about some types of materials, experimental systems and methods used in many studies. Here, indicate whether each material, system or method listed is relevant to your study. If you are not sure if a list item applies to your research, read the appropriate section before selecting a response.

Materials & experimental systems

n/a	Involved in the study	
☐	☒	Antibodies
☐	☒	Eukaryotic cell lines
☒	☐	Palaeontology and archaeology
☒	☐	Animals and other organisms
☒	☐	Human research participants
☒	☐	Clinical data
☒	☐	Dual use research of concern

Methods

n/a	Involved in the study	
☒	☐	ChIP-seq
☒	☐	Flow cytometry
☒	☐	MRI-based neuroimaging

Antibodies

Antibodies used	anti-HNF4a- Abcam, Catalog number: ab41898, Lot number: GR3277145-24<br>anti-OPN: R&D Systems, Catalog Number: AF808, Lot number: BDO0618021<br>anti-ECadherin: R&D Systems, AF478, Lot number: CYG0518081<br>anti-Actin:Anti-actin: Cytoskeleton PHDH1-A, Lot number: 027
Validation	The validation of immunofluorescence is provided on company websites for each product and have been validated and used in our prior publications, reference 15 and 17 in the main manuscript

Eukaryotic cell lines

Policy information about cell lines

Cell line source(s)	The cell line was obtained as gift from Mary C. Wiess's lab, where the cell line is established in reference 58 in the manuscript. The shDLL1 and shJAG1 cell line was created in our lab, described and validated in reference 15 in the main manuscript
Authentication	Reference 58 and 17

Mycoplasma contamination	All cell lines were tested for mycoplasma contamination using PCR, and were negative
Commonly misidentified lines<br>(See ICLAC register)	*Name any commonly misidentified cell lines used in the study and provide a rationale for their use.*
